# Allogeneic Cultivated Limbal Epithelial Sheet Transplantation in Reconstruction of Conjunctival Sac After Chemical and Thermal Burns

**DOI:** 10.3389/fmed.2021.697264

**Published:** 2021-09-06

**Authors:** Ting Wang, Fengmei Shan, Qingjun Zhou, Weiyun Shi, Lixin Xie

**Affiliations:** ^1^Department of Ophthalmology, Eye Hospital of Shandong First Medical University, State Key Laboratory Cultivation Base, Shandong Provincial Key Laboratory of Ophthalmology, Shandong Eye Institute, Shandong First Medical University & Shandong Academy of Medical Sciences, Jinan, China; ^2^Department of Ophthalmology, Qingdao Eye Hospital of Shandong First Medical University, State Key Laboratory Cultivation Base, Shandong Provincial Key Laboratory of Ophthalmology, Shandong Eye Institute, Shandong First Medical University & Shandong Academy of Medical Sciences, Qingdao, China

**Keywords:** chemical and thermal burns, transplantation, symblepharon, allogeneic limbal epithelial cell, reconstructing conjunctival sac

## Abstract

The study aims to evaluate the effect of allogeneic cultivated limbal epithelial cell sheet transplantation (CLET) in reconstructing conjunctival sac for severe symblepharon after chemical and thermal burns. A retrospective, non-comparative case series. Thirty-six eyes (36 patients) underwent CLET for severe symblepharon and conjunctival sac stenosis or atresia. Symblepharon was separated, and pseudopterygium was preserved to replace the palpebral conjunctiva. Allogeneic cultivated limbal epithelial cell sheet using human amniotic membrane as a carrier was transplanted into the recipient's eye to reconstruct the conjunctival sac. The effect of conjunctival sac reconstruction, eye and eyelid movement, ocular surface restitution, and symblepharon recurrence were analyzed after surgery. Symblepharon was completely relieved in 30 of the 36 eyes (83.3%) by a single surgical procedure, with fornix reconstruction, as well as free movement of eye globe and eyelids. Strip-like symblepharon remained in 6 eyes (16.7%) and was completely relieved after the second CLET. Twenty patients without visual function received prostheses 3 months after surgery and the other sixteen patients underwent different corneal transplantation for visual acuity improvement. During the follow-up period, no one had symblepharon recurrence. The transplantation of cultivated allogeneic limbal epithelial sheets offers an effective and safe alternative in the treatment of symblepharon and reconstruction of conjunctival sac in eyes with severe ocular burns, which lays the foundation for subsequent treatments.

## Introduction

Severe chemical and thermal burns can injure the ocular surface. If the limbal and central epithelia are both absent, the neighboring conjunctival epithelial cells will invade the corneal surface, therefore, the surface will be covered with abnormal conjunctiva, called pseudopterygium. In severe cases, symblepharon and conjunctival sac stenosis or atresia may be present. This process is accompanied by chronic inflammation, persistent epithelial defects, stromal scarring, and neovascularization. The key to relieving symblepharon is providing an effective conjunctival substitute. In previous studies, amniotic membrane (AM) (1, 2), autologous nasal mucosa (3), oral mucous membrane (4), as well as cultivated oral mucous membrane (5) with or without a carrier, and autologous cultivated limbal epithelial cells with (6) or without (7) a carrier were used to reconstruct ocular surface. However, most of these methods cannot be used to treat or are ineffective to severe symblepharon in severe burns (8, 9), but limbal stem cell deficiency (LSCD) can. It is known that limited autologous limbal epithelial cells fail to provide enough limbal tissue, and for many patients with severe burns, the contralateral eye is usually involved and could not supply limbal epithelial cells. Oral mucous membrane with abnormal mucus secretion also restricts the use of these methods. Moreover, it is invasive in obtaining autologous tissue. Allogeneic cultivated limbal epithelial transplantation (CLET) for LSCD has been reported, with donor cells obtained non-invasively (10, 11), but its effect on severe symblepharon is unknown. In this study, we aimed to report clinical results of allogeneic CLET in relieving symblepharon and reconstructing conjunctival sac after chemical and thermal burns.

## Materials and Methods

### Patients

This retrospective study was approved by the institutional review board of Shandong Eye Institute. From May 2013 to February 2019, a consecutive series of 36 eyes (36 patients) underwent allogeneic CLET using human AM for severe symblepharon and palpebral fissure deformity from ocular chemical or thermal burns, and each patient completed a follow-up of at least 16 months at Shandong Eye Institute. Among the total, 20 patients (20 eyes) have lost visual function completely (no light perception) before operation and no F-VEP had an analyzable wave pattern preoperatively. Informed consent was obtained from the patients involved in this study. The mean age of the patients was 34.6 ± 15.1 years (range, 7–61 years). The duration between injury and surgery was at least 6 months. The injuries included thermal burns by hot metal in 21 eyes, alkaline burns in 8 eyes, and acidic burns in 7 eyes (Table 1).

**Table 1:** Basic information of the involved patients.

**No**.	**Gender**	**Age (years)**	**Burn**	**Preoperative visual acuity**	**Visual acuity after first CLET**	**Injured eye(s)/treated eye**	**Grading (4)**	**Width**	**Degree of symblepharon recurrence**	**Therapeutic outcomes**
1	male	9	alkaline	NLP	NLP	right/right	2	c	no recurrence	prosthesis
2	male	23	acidic	NLP	NLP	both/right	3	b	no recurrence	prosthesis
3	male	31	acidic	NLP	NLP	left/left	3	c	no recurrence	prosthesis
4	male	39	thermal	HM/BE	FC/10 cm	left/left	3	c	no recurrence	improved appearance and visual acuity
5	male	57	thermal	0.08	0.08	right/right	2	b	no recurrence	improved appearance and visual acuity
6	male	16	thermal	0.1	0.2	right/right	3	c	no recurrence	improved appearance and visual acuity
7	male	55	alkaline	LP	HM/BE	left/left	4	c	2b	improved appearance and visual acuity
8	male	22	thermal	NLP	NLP	right/right	4	c	3a	prosthesis, mild blepharoplasty insufficiency, no recurrence again
9	male	45	thermal	NLP	NLP	left/left	4	b	no recurrence	prosthesis
10	male	41	alkaline	NLP	NLP	both/left	4	b	no recurrence	prosthesis
11	male	27	thermal	NLP	NLP	left/left	2	c	no recurrence	prosthesis
12	male	53	thermal	FC/BE	0.05	right/right	4	b	1a	improved appearance and visual acuity, no recurrence again
13	male	17	thermal	NLP	NLP	left/left	3	b	no recurrence	prosthesis, mild blepharoplasty insufficiency
14	male	54	thermal	NLP	NLP	left/left	3	c	no recurrence	prosthesis
15	male	15	thermal	0.05	0.08	right/right	3	b	no recurrence	improved appearance
16	male	42	acidic	NLP	NLP	left/left	2	c	no recurrence	prosthesis
17	male	40	alkaline	NLP	NLP	right/right	2	c	no recurrence	prosthesis
18	male	45	thermal	0.15	0.12	right/right	4	b	2a	improved appearance, no recurrence again
19	male	55	acidic	NLP	NLP	right/right	3	c	no recurrence	prosthesis
20	male	61	alkaline	NLP	NLP	right/right	4	c	2a	prosthesis, no recurrence again
21	female	48	alkaline	NLP	NLP	both/left	3	b	no recurrence	prosthesis
22	male	31	thermal	NLP	NLP	both/left	3	c	no recurrence	prosthesis
23	male	42	thermal	FC/10 cm	FC/20 cm	both/left	3	b	no recurrence	improved appearance
24	male	43	thermal	HM/50 cm	FC/20 cm	right/right	3	b	no recurrence	improved appearance and visual acuity
25	male	19	thermal	0.04	0.04	right/right	4	b	no recurrence	improved appearance
26	male	32	alkaline	FC/BE	FC/30 cm	left/left	2	c	no recurrence	improved appearance
27	male	7	thermal	NLP	NLP	left/left	3	b	no recurrence	prosthesis
28	female	46	thermal	NLP	NLP	left/left	3	b	no recurrence	prosthesis
29	female	21	acidic	NLP	NLP	right/right	3	c	no recurrence	prosthesis
30	male	20	alkaline	NLP	NLP	right/right	3	a	no recurrence	prosthesis
31	female	53	thermal	NLP	NLP	left/left	3	c	no recurrence	prosthesis, mild blepharoplasty insufficiency
32	female	20	thermal	0.08	0.1	right/right	4	b	no recurrence	improved appearance
33	male	11	thermal	0.02	0.3	right/right	3	c	no recurrence	improved appearance and visual acuity
34	male	26	acidic	0.05	0.2	both/right	2	c	no recurrence	improved appearance and visual acuity
35	male	16	thermal	0.02	0.5	left/left	4	b	2a	improved appearance and visual acuity
36	male	49	acidic	0.05	0.2	both/right	2	c	no recurrence	improved appearance and visual acuity

The eligible eyes in the study met the following inclusion criteria: (1) All eyes had an extensive symblepharon of at least 2 quarters with conjunctival sac contraction, and the pseudopterygium encroaches onto the cornea, with limitation of eye movement and palpebral fissure deformity; (2) intraocular pressure <22 mmHg without symptoms; (3) no obvious changes of vitreous body and retina, as detected by B-scan ultrasound; (4) allogeneic CLET was performed at more than 6 months after burns.

### Cultivation of Limbal Epithelium

Human immunodeficiency viruses 1 and 2 syphilis, hepatitis B and C, and were negative in all donors. Limbal tissue was cut from fresh donor eyeballs, separated into pieces, and inoculated on denuded AM on the transwell insert as an explant culture. Then the surgeon transferred the transwell inserts into a cell culture plate pre-seeded with mitomycin C-inactivated NIH 3T3 feeder cells, after which the incubation was performed until confluence and stratification for up to 5 days, and the medium was changed every 48 h. The cell sheets, which were approximately 2 cm × 2 cm with 3 to 5 layers of stratification on the AM and basal column-shaped cells and superficial flattened scale-like cells, were used for transplantation (12, 13).

### Surgical Techniques

The surgery was guided by optical coherence tomography (OCT). Firstly, the pseudopterygium was peeled off from the cornea and sclera. The symblepharon was separated completely until the eye globe could be moved freely by the surgeon during the procedure. Secondly, the surgeon separated the fibrous tissues of the pseudopterygium with blunt dissection until exposing the sclera. We receded the remaining pseudopterygium as the replacement of palpebral conjunctiva (14) and sutured it inside the surface of the eyelid with 10–0 nylon sutures. The human AM with the cultivated allogeneic limbal epithelial sheet was placed on the corneal stroma, sclera, fornix, and palpebral conjunctiva with the epithelial side oriented upward and sutured with interrupted 10-0 nylon sutures. At the end of the surgery, the surgeon placed a therapeutic soft contact lens on the surface of the treated eyes.

### Postoperative Treatment

Intravenous methylprednisolone (2 mg/kg) was administered every day for 3 days. Oral prednisolone (1 mg/kg) was given daily at the beginning of the medication treatment and then tapered over about 3 months. Autologous serum eye drops, with 0.02 mg of dexamethasone and 0.1 mg of tobramycin per milliliter of serum in them, were used every 2 h for the first week, and 0.02% fluorometholone eye drops were administered 4 times daily for the next 2 weeks. After the corneal epithelial was cured, 1% cyclosporine A eye drops were administered 4 times daily. Tobramycin ophthalmic ointment was given every night. This therapy was adjusted according to the patients' clinical status after 3 weeks. The patients were observed every day during the first week after the transplantation, weekly during the next 1 month, monthly in the next 3 months, and 3 months thereafter.

Intravenous methylprednisolone (2 mg/kg) was administered every day for 7 days in the cases that immune rejection occurred. Oral prednisolone (1 mg/kg) was given daily at the beginning and tapered for 2 months. Tobramycin and dexamethasone eyedrops were given every 2 h for the first 3 days and tapered to 4 times daily for the next 3 weeks. Then, 0.02% fluorometholone eye drops were administered 4 times every day. At the same time, 1% cyclosporine eye drops were applied 4 times daily. Tobramycin and dexamethasone ophthalmic ointment was given every night for 1 month and twice weekly during taper.

Three months after surgery, a suitable ocular prosthesis was implanted in 20 patients who lost visual function, and ofloxacin 0.3% ophthalmic solution was administered 4 times daily for 2 weeks. And at the same time, cyclosporine A 1% eye drops were given twice daily for a long period of time in all patients.

### Outcome Measures

After a detailed explanation, the patient's history, symptoms, onset time, frequency of immune rejection, preoperative and postoperative photographs were collected. Slit-lamp microscopy was performed. Symblepharon recurrence, immune rejection, eye globe movement, prosthesis suitability, and eyelid activity were evaluated.

## Results

**Figure 1:**
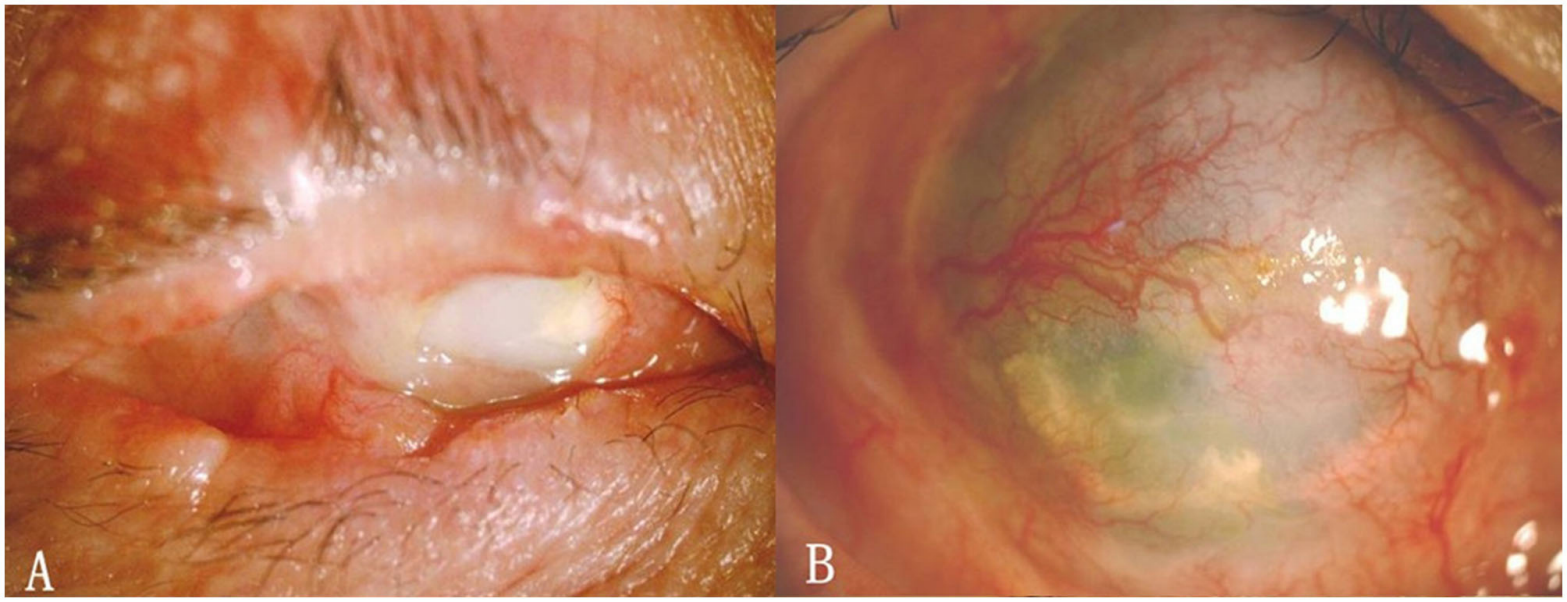
**(A)** Extensive symblepharon with conjunctival sac atresia. **(B)** Symblepharon was completely released 9 months after allogeneic CLET (A and B are from the same patient).

**Figure 2:**
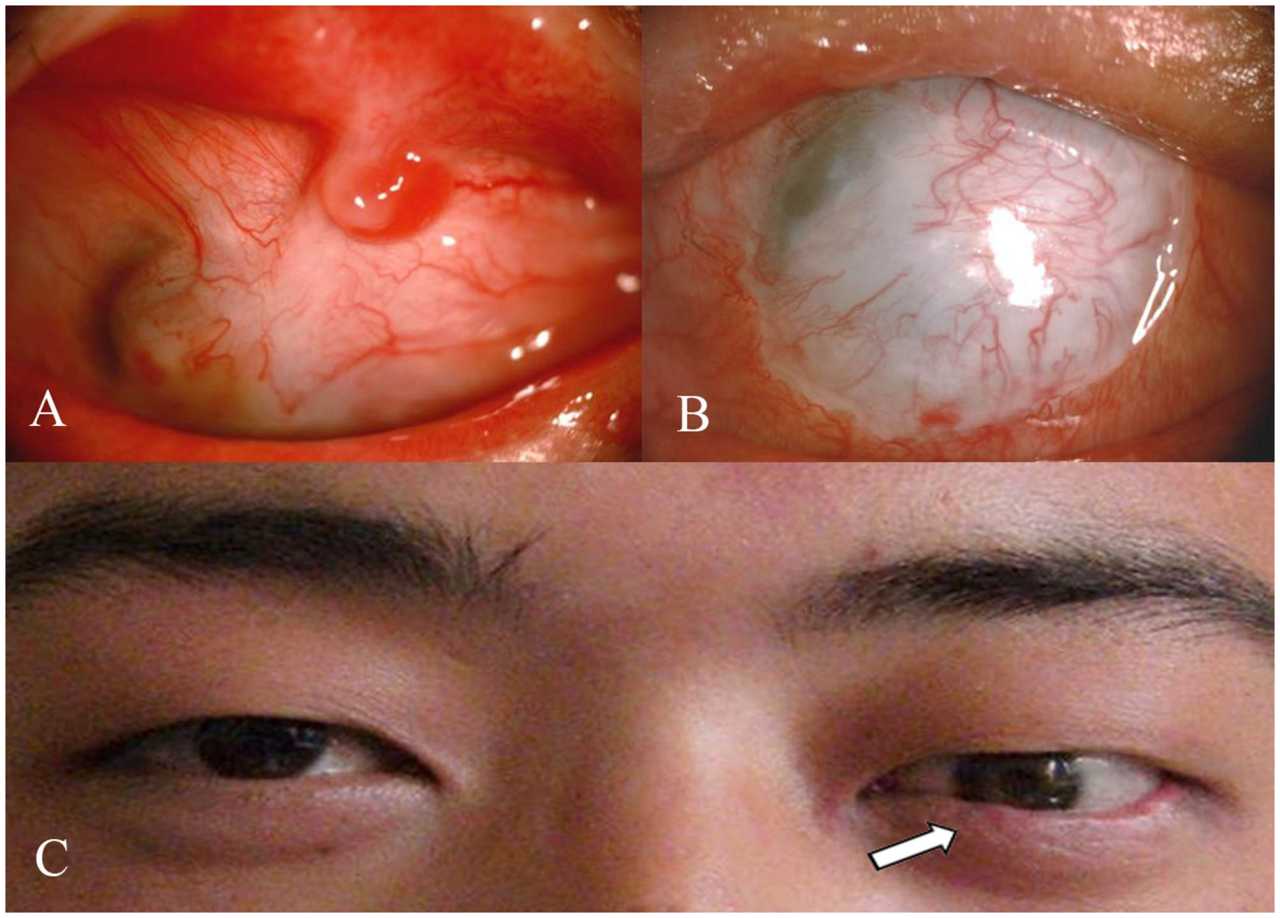
**(A)** Strip-like symblepharon remained after the first CLET. **(B)** Symblepharon was completely released after the second allogeneic CLET. **(C)** A suitable prosthesis was implanted in the eye that has lost visual function, and the appearance was obviously improved.

Symblepharon was completely released in 30 eyes (83.3%) 3 months after a single surgical procedure (Figure 1). Strip-like symblepharon, <3 mm wide (Figure 2A), remained in 6 eyes (16.7%). Further symblepharon separate on combined with second allogeneic CLET was performed (Figure 2B), and a suitable prosthesis was implanted in the 20 patients who lost visual function (Figure 2C). Over a follow-up period of 18 months (mean, 17.4 ± 3.9 months), no symblepharon recurred in all 36 patients. Three patients (8.3%) with mild palpebral fissure dysraphism suffered immune rejection within 6 months after surgery, restored a stable ocular surface after 7 days of antirejection therapy.

### Reconstruction of Conjunctival Sac

Thirty patients (eyes) could move freely with the smooth ocular surface, the eyelids could blink normally. The incidence rate of Symblepharon after allogeneic CLET was 16.7% (6 eyes of 6 patients) in the first 3 months after surgery. The original injuries included thermal burns in 4 eyes (4 patients) and chemical burns in 2 eyes (2 patients). Six patients (6 eyes) were found strip-like symblepharon of 1–2 mm extent, at 1–3 months after surgery. Follow up 3 months, the wide of symblepharon increased to about 3 mm in one patient, other patients had no distinguishable change, the fornix depth in other parts was not shallow gradually. Further symblepharon separation combined with second allogeneic CLET was performed 2–3 months after symblepharon occurred. Follow up to now, no symblepharon recurrence.

### Immune Rejection

3 eyes of 3 patients suffered immune rejection within 6 months after surgery, 1 patient had redness, irritation, photophobia 3 days after stopped taking 1% cyclosporine for 8 days at 4 months after surgery. The clinical manifestation was visual acuity decreased from 0.1 to 0.02, conjunctival congestion, limbal hyperplastic and dilatational vessels, which extended up to the corneal stroma, corneal edema, opacification, epithelial asperity, no rejection line, epithelial defects, keratic precipitates, or anterior chamber reactions. One patient (male, 40 years) with mild palpebral fissure dysraphism suffered immune rejection 1.5 months after surgery, who was burned by alkali severely. He had irritation, Palpebral hypertrophy, ocular surface congestion widely, without secretion and symblepharon recurrence. one patient (male, 55 years) was asymptomatic and diagnosed during the routine follow-up at 2 months after the second surgery, who was also burned by alkali, had severe symblepharon and conjunctival sac atresia. He had the same manifestation as the previous patient. All the 3 patients restored a stable ocular surface after 7 days of antirejection therapy, and the globe and eyelids could move freely without symblepharon recurrence.

### Improve Appearance and Visual Function Recovery

Two to three months later, the best-corrected visual acuity (BCVA), intraocular pressure, ocular B-ultrasound and F-VEP were examined. Among the total, 20 patients (20 eyes) have lost visual function completely due to abnormal eye pressure. 3–4 months after surgery, 2 patients with recurrent symblepharon underwent second allogeneic CLET and further symblepharon separation. For follow-up at 3 months, no symblepharon recurred; with a suitable prosthesis, the appearance of these 20 patients was improved.

**Figure 3:**
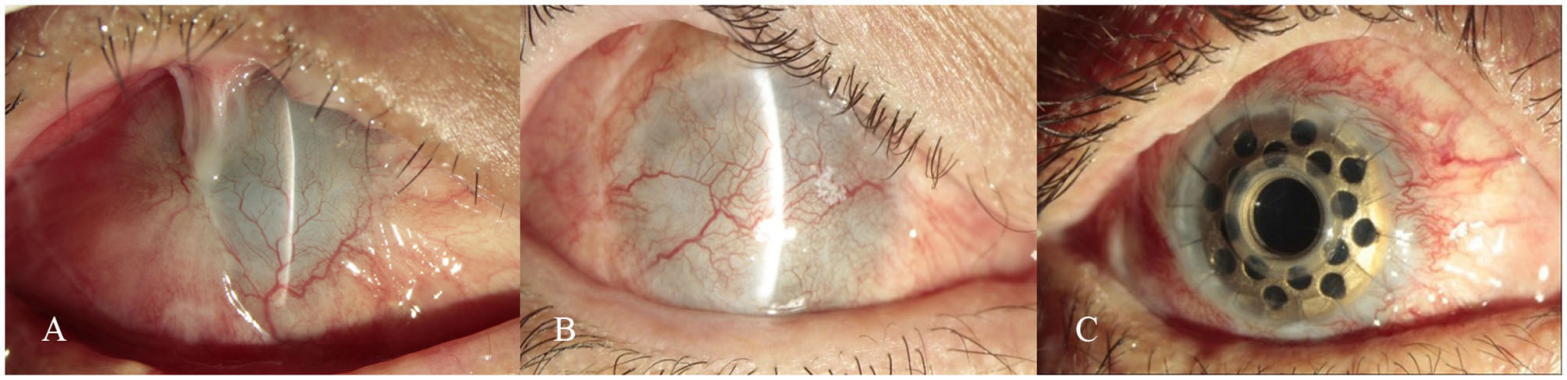
**(A)** Extensive pseudopterygium covered half of the cornea, with severe symblepharon. **(B)** Symblepharon was completely released at 10 months after allogeneic CLET. **(C)** The best-corrected visual acuity (BCVA) had improved to 0.6 with a stable ocular surface at 6 months after implanting collar-button-shaped Keratoprosthesis.

The other 16 patients (16 eyes) had varying degrees of poor BCVA, and the intraocular pressure was within the normal range, F-VEP had a significant wave pattern, vitreous body with mild opacity in B-ultrasound, denote no obvious abnormality. Over the follow-up period, lacrimal secretion, with or without symblepharon recurrence and immune rejection was observed. More than 6 months after CLET, lamellar keratoplasty (LKP), penetrating keratoplasty (PKP) or transplantation with keratoprosthesis was selected and performed according to the recovery of tears, the degree of limbal stem cells deficiency, and the depth of corneal opacity of each patient to improve the vision. For patients whose Schirmer's test reached 5 mm in follow up, LKP or PKP can be performed; for patients with full-thickness corneal opacity or poor corneal endothelial function, PKP can be performed; if the patient's Schirmer's test reached 2 mm but <5 mm and the patient has a high risk of rejection (severe limbal stem cell deficiencies), transplantation with keratoprosthesis (Guangdong Brilliant Vision Biotechnology Co., Ltd.) can be carried out (Figure 3).

## Discussion

Severe ocular burns can destroy conjunctiva, limbal stem cells, and cornea, inevitably resulting in severe symblepharon with limitation of eye movement and ocular deformity. Autologous conjunctival transplantation was effective for mild symblepharon, but the application of the method is limited because autologous limbal epithelial cells cannot be removed too much from the fellow eye, or the fellow eye was also involved in burns and can't provide limbal epithelial cells. Moreover, obtaining autologous tissue is invasive and painful. Oral mucous membrane, as well as cultivated oral mucous membrane, can be used to reconstruct ocular surface. Cultivated autologous oral mucous membrane (5) and autologous cultivated limbal epithelial cells (6, 7, 15) were used for conjunctival substitutes in recent years. But oral mucous membrane is different from the conjunctiva, with the defect of opacity, shrink, abnormal mucus secretion and unsatisfactory cosmetic results, epithelial failure, and corneal melting (16, 17). Unfortunately, most of these methods cannot be used for the treatment of severe symblepharon. As we all know, the extensive use of AM has greatly improved the prognosis of acute ocular burns, but AM was a basilar membrane without epithelium. Therefore, it could not substitute for conjunctiva completely and symblepharon recurrences occurred in many cases. A severe symblepharon is often accompanied by eyelid malformation, large-scale conjunctival deficiency, limbal stem cell destruction, and corneal opacification, so a single approach cannot resolve all the problems. Allogeneic CLET has been found to be effective for the treatment of total LSCD. Allogeneic cultivated limbal epithelial cells as a substitute for corneal epithelium can treat corneal neovascu In this study, we first reported that allogeneic CLET is an effective surgical method for symblepharon repair and can maintain a deep fornix.

In our study, the patients with severe symblepharon were treated by CLET to reconstruct the conjunctival sac and improve the activity of the eye globe and eyelids, and a good cosmetic effect was achieved. A few patients suffered strip-shaped symblepharon after the first surgery, which may be related to severe symblepharon, insufficient separation, inadequate allogeneic stem cells, and amniotic membrane contracture. All these patients had severe symblepharon involving more than 3 quadrants before surgery, and the cornea completely adhered to the pseudopterygium. For these patients, it was not difficult to perform a second allogeneic CLET, and favorable results were achieved.

During the surgery, the first step was peeling off the pseudopterygium from the cornea, and then it was used to reconstruct conjunctiva palpebrae. The operation was designed in this way for the following reasons: (1) tissues that are discarded in conventional surgical methods can be used effectively; (2) preservation of pseudopterygium can obviously diminish the conjunctival deficiency area, requiring smaller size of the cultivated graft to reestablish ocular surface; (3) as an autologous tissue, pseudopterygium does not lead to immune rejection.

For the patients who lost visual function, implantation of a suitable prosthesis could achieve satisfactory cosmetic results after allogeneic CLET. For the patients who still have visual function, second corneal transplantation can give them a chance to increase vision (7).

To ensure a successful surgery, we upheld the principles as follows: (1) separate symblepharon completely, expose wound surface sufficiently, and clear up the scar tissue as much as possible; (2) try to preserve the conjunctival surface of pseudopterygium; (3) avoid fluid between the amniotic membrane with cultured epithelial cells and the underlying ocular surface; (4) the graft cell sheet is big enough compared with the wound surface, and if necessary 2 pieces of cell sheets or more are used; (5) a highly permeable contact lens is fixed like a bandage on the eye to improve the survival rate of graft.

Our study had several limitations. First, it was a retrospective study. Further studies with a randomized design, a larger sample size, and a long-term follow-up are needed. Second, a few patients developed immune rejection after surgery, which may be attributed to the inflammation induced by palpebral fissure dysraphism. Fortunately, the rejection was controlled after anti-rejection therapy, with no symblepharon recurrence. This study just introduced a novel method in reconstructing conjunctival sac for severe symblepharon. We did not report further treatment after the following keratoplasty. The therapeutic effect on severe symblepharon was the focus of this study.

In summary, allogeneic CLET can successfully relieve symblepharon and reconstruct conjunctival sac in eyes with severe ocular burns, not only improving the appearance of late-stage patients but also laying a foundation for further improvement of eyesight for some patients.

## Data Availability Statement

The original contributions presented in the study are included in the article/supplementary material, further inquiries can be directed to the corresponding author/s.

## Ethics Statement

The studies involving human participants were reviewed and approved by the institutional review board of Shandong Eye Institute. Written informed consent to participate in this study was provided by the participants' legal guardian/next of kin. Written informed consent was obtained from the individuals for the publication of any potentially identifiable images or data included in this article.

## Author Contributions

TW, WS, and LX contributed to conception and design of the study. TW and FS organized the database. QZ performed the statistical analysis. TW wrote the first draft of the manuscript. FS, QZ, WS, and LX wrote sections of the manuscript. All authors contributed to the article and approved the submitted version.

## Funding

This study was supported by the Shandong Provincial Natural Science Foundation (ZR2019MH135), National Natural Science Foundation Regional Innovation and Development Joint Fund (U20A20386), Young Taishan Scholars (tsqn201909188), and Academic Promotion Programme of Shandong First Medical University (2020RC004).

## Conflict of Interest

The authors declare that the research was conducted in the absence of any commercial or financial relationships that could be construed as a potential conflict of interest.

## Publisher's Note

All claims expressed in this article are solely those of the authors and do not necessarily represent those of their affiliated organizations, or those of the publisher, the editors and the reviewers. Any product that may be evaluated in this article, or claim that may be made by its manufacturer, is not guaranteed or endorsed by the publisher.
